# Naringenin suppresses BEAS-2B-derived extracellular vesicular cargoes disorder caused by cigarette smoke extract thereby inhibiting M1 macrophage polarization

**DOI:** 10.3389/fimmu.2022.930476

**Published:** 2022-07-18

**Authors:** Zhen Chen, Hao Wu, Weiyang Fan, Jiashuo Zhang, Yue Yao, Weiwei Su, Yonggang Wang, Peibo Li

**Affiliations:** Guangdong Engineering and Technology Research Center for Quality and Efficacy Re-evaluation of Post-marketed Traditional Chinese Medicine (TCM), State Key Laboratory of Biocontrol, Guangdong Provincial Key Laboratory of Plant Resources, School of Life Sciences, Sun Yat-sen University, Guangzhou, China

**Keywords:** cigarette smoke, naringenin, exosome, macrophage polarization, epithelium-macrophage crosstalk

## Abstract

Extracellular vesicles (EVs)-mediated epithelium-macrophage crosstalk has been proved to maintain lung homeostasis in cigarette smoke-induced lung diseases such as chronic obstructive pulmonary disease (COPD). In our previous study, we found that EVs derived from cigarette smoke extract (CSE) treated BEAS-2B promoted M1 macrophage polarization, which probably accelerated the development of inflammatory responses. Naringenin has been proved to suppress M1 macrophage polarization, but whether naringenin regulates macrophage polarization mediated by EVs has not been reported. In this study, we firstly found that EVs derived from naringenin and CSE co-treated BEAS-2B significantly inhibited the expression of CD86 and CD80 and the secretion of tumor necrosis factor (TNF)-α, interleukin (IL)-6, IL-1β, inducible nitric oxide synthase (iNOS), and IL-12 in macrophage induced by EVs derived from CSE-treated BEAS-2B. Further research revealed that naringenin downregulated BEAS-2B-derived EVs miR-21-3p which targeted phosphatase and tensin homolog deleted on chromosome ten/protein kinase B (PTEN/AKT) cascade in macrophages and then suppressed M1 macrophage polarization. Subsequent proteomics suggested that naringenin decreased BEAS-2B-derived EVs poly ADP-ribose polymerase (PARP)1 expression thereby suppressing M1 macrophage polarization probably. Our study provides novel pharmacological references for the mechanism of naringenin in the treatment of cigarette smoke-induced lung inflammatory diseases.

## Introduction

Long-term exposure to cigarette smoke predisposes to and accelerates the development of inflammation in chronic lung diseases such as chronic obstructive pulmonary disease (COPD) and asthma (1). Extracellular vesicles (EVs), novel intercellular communication tools, contain bioactive molecules such as nucleic acids, proteins, and lipids (2). Recently, EVs-mediated epithelium-macrophage crosstalk was proved to maintain lung homeostasis, whereas aberrant crosstalk probably led to immune imbalance and inflammatory response in lung diseases (3). Excessive accumulation of M1 macrophages at inflammatory sites can lead to exacerbated tissue damage and more severe inflammatory storms (4). In our previous study, we found that cigarette smoke extract (CSE) upregulated the human bronchial epithelial cell line (BEAS-2B)-derived EVs miR-21-3p and miR-27b-3p thereby promoting M1 macrophage polarization (5).

Naringenin, a natural flavanone, is abundantly present in *citrus* fruits and has shown strongly anti-inflammation activities in multiple *in vitro* and *in vivo* studies of lung diseases such as COPD, asthma, lung fibrosis, and acute lung injury (6). Furthermore, Karuppagounder et al. (7) found that naringenin could suppress M1 macrophage polarization thereby ameliorating skin inflammation in atopic dermatitis mice. However, whether naringenin can affect the extracellular vesicular cargoes profiles derived from airway epithelium and then regulate macrophage polarization has not been reported.

Consequently, in this study, we investigated whether naringenin could downregulate the EVs miR-21-3p or miR-27b-3p levels derived from airway epithelium thereby regulating M1 macrophage polarization. Moreover, considering that contained proteins were also critical for EVs to exert biological functions, we employed proteomics to explore whether naringenin altered protein expression profiles of EVs and then regulated M1 macrophage polarization. Our findings provided more comprehensive pharmacological evidence for naringenin in the treatment of cigarette smoke-induced lung diseases.

## Materials and methods

### Chemicals and reagents

The BEAS-2B cells were obtained from iCell (Shanghai, China), and the murine alveolar macrophage cell line (MH-S) was purchased from Procell (Wuhan, China). The murine leukemia macrophage cell line (RAW264.7) and the human myeloid leukemia mononuclear cell line (THP-1) were obtained from EK-Bioscience (Shanghai, China). High glucose dulbecco’s modified eagle medium (DMEM) was acquired from HyClone (Logan, UT, USA). Roswell park memorial institute (RPMI) 1640 medium was purchased from Gibco (Grand Island, NY, USA). Fetal bovine serum was purchased from Corning (Auckland, New Zealand) and penicillin/streptomycin was obtained from Gibco. Naringenin, dimethyl sulfoxide (DMSO), and phorbol ester (PMA) were obtained from Sigma-Aldrich (St. Louis, MO, USA). Specific phosphatase and tensin homolog deleted on chromosome ten (PTEN) inhibitor (SF1670) and poly ADP-ribose polymerase (PARP)1 inhibitor (AG14361) were acquired from MCE (Monmouth Junction, NJ, USA).

### CSE preparation

CSE was prepared as previously reported (8). Briefly, the smoke of three commercial cigarettes (Cocopalm, Guangzhou, China) was pumped into 5 ml warm medium by a bt100-2j peristaltic pump (LongerPump, Baoding, China). Before administration to cells, the CSE solution is needed to be filtered through 0.22-μm-pore-size sterilizing filters. The solution was diluted 10-fold, after which the absorbance was measured at 320 nm. If the absorbance approaches 0.8, the resultant CSE solution was regarded as 100% CSE, which was diluted with DMEM medium before being used.

### Cell culture and treatment

BEAS-2B cells were cultured in high glucose DMEM plus 10% fetal bovine serum and 1% penicillin/streptomycin at 37°C and 5% CO_2_. THP-1 monocytes were treated with 100 ng/ml PMA for 48 h and differentiated into M0 macrophages on day 3. RAW264.7, MH-S, and THP-1 macrophages were cultured in RPMI 1640 medium plus 10% fetal bovine serum and 1% penicillin/streptomycin at 37°C and 5% CO_2_. When 80-90% confluence was reached, BEAS-2B cells were incubated with EVs-free DMEM (100,000×g overnight to remove the EVs in fetal bovine serum) at 37°C and 5% CO_2_ for 24 h. Then, the supernatants were harvested to isolate EVs.

### EVs extraction

EVs were isolated as described previously (9). Briefly, the supernatants were centrifuged at 300×g and 2,000×g for 10 min, respectively. Next, the supernatants were centrifuged at 10,000×g for 30 min. Then, we filtered the harvested supernatants with 0.22-μm filters. Subsequently, ultracentrifuge (Beckman, Brea, CA, USA) was utilized for the centrifugation of supernatants at 100,000×g for 70 min. Finally, the required pellets were resuspended with PBS before being kept at -80°C.

The abbreviations of each group of EVs are as follows: Control-EVs: BEAS-2B cells were treated without any specific treatment, and supernatants were harvested after 24 h for EVs isolation; CSE-EVs: the cells were treated with 7.5% CSE and supernatants were harvested for EVs isolation; CSE+NRG-EVs: the cells were co-treated with 7.5% CSE and 100 µM naringenin and supernatants were harvested for EVs isolation; CSE+DMSO-EVs: the cells were co-treated with 7.5% CSE and 0.1% DMSO (vehicle of naringenin) and supernatants were harvested for EVs isolation.

### EVs identification

Isolated EVs were imaged utilizing the JEM-1400Flash transmission electronic microscopy (TEM) (JEOL Ltd., Japan) at a voltage of 120 kV. The size distributions of EVs were determined by nanoparticle tracking analysis (NTA) using a NanoSight-NS300 (Malvern Instrument, Malvern, UK). Total protein concentrations of EVs were measured using BCA Protein Assay Kits (Beyotime, Nanjing, China) according to the manufacturer’s protocols. Marker proteins of EVs were detected by western blotting. ALIX (1:1000, ab275377, Abcam), TSG101 (1:1000, ab133586, Abcam), CD9 (1:2000, ab50249, Abcam) were used.

### High-performance liquid chromatography with tandem mass spectrometry

Naringenin in CSE+NRG-EVs was detected by HPLC-MS/MS using a 1200SL HPLC-6410 QQQ liquid chromatograph-mass spectrometer (Agilent, Santa Clara, CA, USA). EVs samples were prepared in acetonitrile. Naringenin at a concentration of 200 ng/ml was prepared as a reference standard. The chromatographic separation was achieved on an Agilent Poroshell 120 EC-C18 column (3.0 × 30 mm, 2.7 μm) at 45°C. Mobile phase: 0.1% methanol and water (45:50, v/v, both containing φ = 0.1% formic acid), with a flow rate of 400 μl/min. Mass spectrometric analysis was performed on the negative ion multiple reaction monitoring mode, with Capillary 4000 V, Drying Gas 10 L/min, Neb Pressure 25 psi, Gas Temp 350°C. For naringenin, the collision energy was 12 V and the multiple reaction monitoring modes with a precursor to product qualifier transition m/z was 270.9/150.7.

### Detection of uptake of EVs by macrophages

EVs extracted from the supernatants of BEAS-2B cells were labeled with PKH67 green fluorescent (Sigma-Aldrich) according to the experimental procedures described by the previous study (10). Briefly, 400 μl EVs were mixed with 1.6 ml Diluent C and 8 μl PKH67 dye for 5 min at room temperature, and 1% bovine serum albumin (BSA) was added to terminate the dying process. PKH67-labeled EVs were recollected at 100,000×g for 70 min at 4°C. PKH67-labeled EVs or the PKH67-PBS control were separately incubated for 12 h with macrophages seeded on confocal dishes. Nuclei of macrophages were stained with 5 μg/ml DAPI (Sigma-Aldrich) for 10 min at room temperature and then observed under an SP8X laser scanning confocal microscope (Leica, Wetzlar, Germany).

### Flow cytometry

The polarization-related surface markers of RAW264.7, MH-S, and THP-1 macrophages were detected by flow cytometry. In our previous study (5), we found that 10 μg/ml CSE-EVs could significantly promote M1 polarization of RAW264.7 macrophages, thus we treated macrophages (5×10^4^ cells/cm^2^) with 10 μg/mL EVs in EVs-free RPMI 1640 mediums for 24 h in this study. For positive control, 2 μM SF1670 and 1 μM AG14361 were used to inhibit PTEN and PAPR1 expression of THP-1 cells, respectively. Then, the cells were collected and stained with the following antibodies on ice for 20 min in the dark. APC anti-mouse CD86, APC anti-mouse CD80, APC anti-human CD11b, PE anti-human CD86, and PE anti-human CD80 (Biolegend, San Diego, CA, USA) were used in this procedure. After being washed twice with PBS, cells were fixed with fixation buffer and then analyzed by flow cytometry (CytoFLEX, Beckman).

### Enzyme-linked immunosorbent assay

Cell supernatants were collected from RAW264.7 and THP-1 macrophages and centrifuged at 3000 rpm at 4°C for 20 min. The concentrations of tumor necrosis factor (TNF)-α, interleukin (IL)-6, IL-1β, inducible nitric oxide synthase (iNOS), and IL-12 were determined with ELISA Assay Kits (MeiMian, Wuhan, China) according to the manufacturer’s instructions. All samples were measured at 450 nm wavelength by an Epoch-2 microplate reader (BioTek, Winooski, VT, USA).

### RNA isolation and quantitative real-time PCR

Total RNA from THP-1 macrophages was extracted using TRIzol (Invitrogen, Carlsbad, NY, USA). Total EVs RNA was isolated using Cell Culture Media Exosome Purification Kits (Norgen Biotek, Canada) following the manufacturer’s instructions. The RNA quality and quantity were determined using the NanoDrop 2000c (Thermo Fisher Scientific, MA, USA). Real-time PCR was performed with GoTaq^®^ qPCR Master Mix (Promega, Madison, WI, USA) using a LightCycler 480 System (Roche, Mannheim, Germany). The primer sequences are listed in **Supplementary 1**. The relative expression of the target mRNA was normalized to GAPDH and the target miRNA was normalized to U6. All relative changes in gene expression were analyzed by the 2^−ΔΔCT^ method.

### Western blotting

THP-1 macrophages were seeded in 6-well plates at 5×10^4^ cells/cm^2^ and treated with 10 μg/mL EVs for 24 h. Then, they were lysed by RIPA buffer containing protease inhibitors (Beyotime, Shanghai, China). Protein lyses were collected after centrifuging at 16,000 rpm for 30 min and the concentration was determined by BCA Protein Assay Kits. Then, proteins were loaded on 10% SDS-polyacrylamide gels and transferred to polyvinylidene difluoride membranes. The membranes were blocked with 5% fat-free milk for 2 h at room temperature and incubated with specific antibodies overnight at 4°C. PTEN (1:1000, 9188T, CST), AKT (1:1000, 4691T, CST), p-AKT (1:1000, 4060T, CST), and GAPDH (1:10000, ab8245, Abcam) were used in this procedure. After being washed, the membranes were incubated with corresponding HRP-conjugated secondary antibodies at room temperature for 1 h. The bands were visualized by a chemiluminescence imaging system (Tanon, Shanghai, China). ImageJ software (NIH, Bethesda, MD, USA) was used to quantify the integrated density of bands.

### Protein filter-aided sample preparation of EVs

EVs were isolated with SDT buffer (4% SDS, 100 mM Tris-HCl, PH 7.6) and the proteins of EVs were quantified with the BCA Protein Assay Kit. Two hundred μg of proteins for each sample were reduced with 50 mM dithiothreitol and repeated ultrafiltration using a UA buffer (8 M Urea, 150 mM Tris-HCl, pH 8.5). Then 100 μl iodoacetamide was added to block reduced cysteine residues and the filters were washed with 100 μl UA buffer three times and then 100 μl 25 mM NH_4_HCO_3_ buffer twice. Finally, the protein suspensions were digested with 4 μg trypsin in NH_4_HCO_3_ buffer overnight at 37°C, and the resulting peptides were collected as a filtrate.

### Nanoscale liquid chromatography coupled to tandem mass spectrometry

Samples were analyzed on a nanoElute (Bruker, Bremen, Germany) coupled to a timsTOF Pro (Bruker, Bremen, Germany) equipped with a CaptiveSpray source. Peptides were separated on an analytical column, 1.6μm C18 beads with a packed emitter tip (IonOpticks, Australia). The column was equilibrated using 4 column volumes before loading the sample in 100% buffer A (99.9% MilliQ water, 0.1% FA). Samples were separated at 300 nl/min using a linear gradient and the timsTOF Pro was operated in PASEF mode. The raw data were analyzed using MaxQuant software version 1.6.14.0. The cutoff of the false discovery rate for peptide and protein identification was set to 0.01. Protein abundance was calculated based on the normalized spectral protein intensity. Proteins which Fold change > 2 or < 0.5 and *p*-value < 0.05 were considered to be differentially expressed proteins.

### Bioinformatics analysis

Bioinformatics analyses for differentially expressed EVs proteins in this study were performed using the OmicStudio tools at https://www.omicstudio.cn/tool. The heat map was performed using the Advanced Heatmap Plots tool to visualize all of the differentially expressed proteins. The annotation from Gene Ontology (GO) terms to proteins was completed by Blast2GO Command-Line. Then further improvement of annotation and connection between GO terms were carried out by ANNEX. Pathway analysis was performed using the KEGG database. Fisher’s Exact Test was used to identify the significantly enriched GO terms and pathways by comparing the number of differentially expressed proteins and total proteins.

### Statistical analysis

Experiment results were expressed as the mean ± SD of at least 3 independent experiments and statistical analyses were performed using GraphPad Prism 8 software. Differences between groups was assessed by unpaired two-tailed Student’s t-test for comparison of two groups and one-way analysis of variance (ANOVA) followed by Dunnett’s test for multiple groups. A *p* < 0.05 was considered the minimum level of statistical significance.

## Results

### The characterization of BEAS-2B-derived EVs

**Figure 1:**
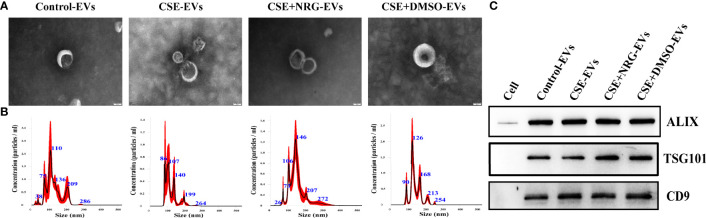
The characterization of BEAS-2B-derived EVs. **(A)** The morphology of EVs imaged by transmission electronic microscopy. Scale bar = 50 nm. **(B)** The size distribution and concentration of EVs were measured by nanoparticle tracking analysis. **(C)** ALIX, TSG101, and CD9 were detected by western blotting. BEAS-2B cellular total protein was used as a negative control.

EVs were isolated from the supernatants of BEAS-2B cells. TEM, NTA, and western blotting were carried out to identify the EVs. The morphology of EVs was visualized using TEM. We observed that the purified EVs have cup or spherical shapes with membrane vesicle structures (**Figure 1A**). NTA analysis showed mere peaks of EVs at approximately 120 nm with mean diameters within 150 nm (**Figure 1B**). The concentrations of EVs were approximately ranging from 5.7 × 10^8^ to 6.8 × 10^8^ particles/ml. Western blotting illustrated that ALIX, TSG101, and CD9 were enriched in the EVs pool but not in the BEAS-2B cells pool (**Figure 1C**).

### CSE+NRG-EVs almost did not contain naringenin

**Figure 2:**
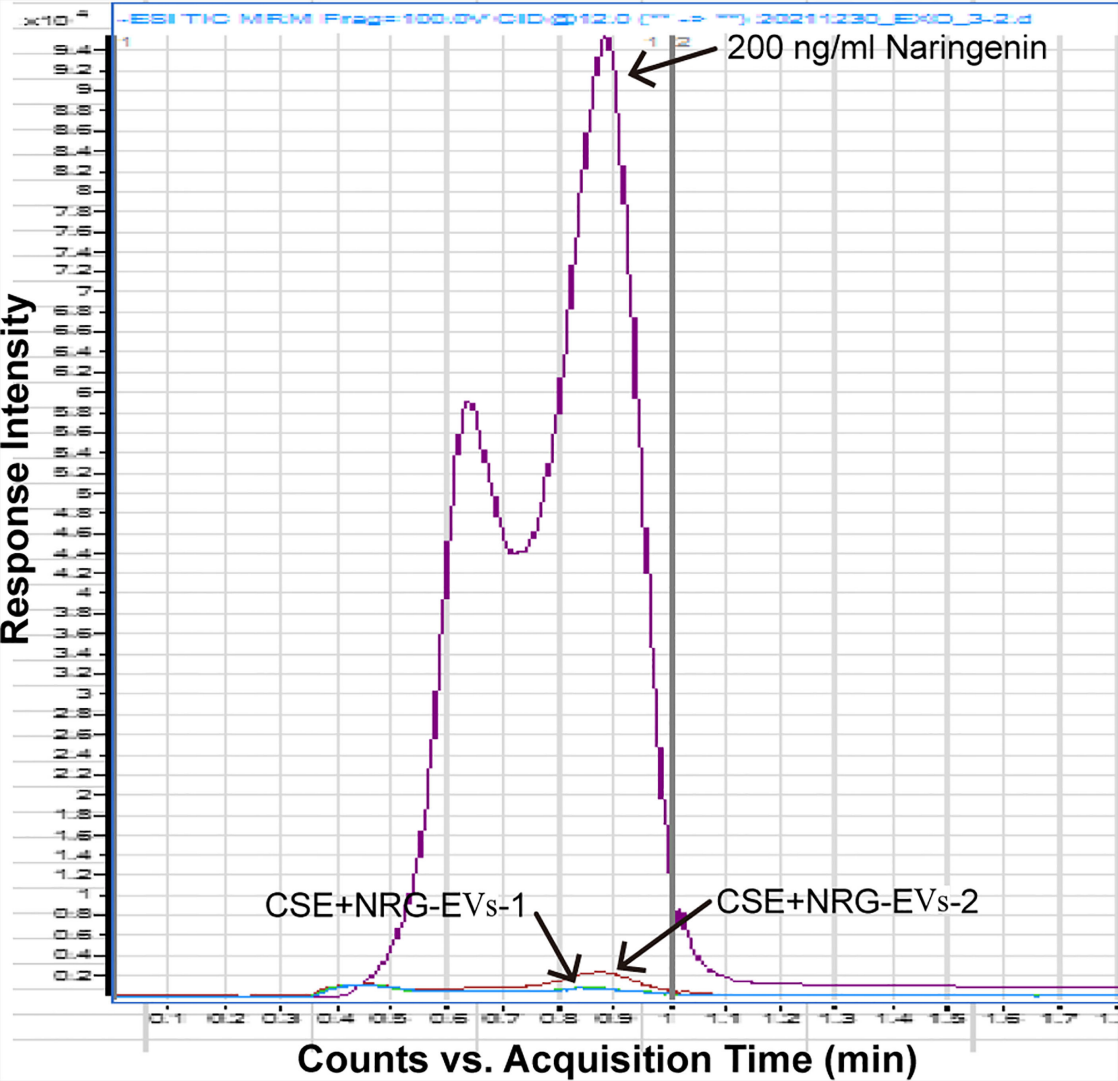
Detection of naringenin in CSE+NRG-EVs by high-performance liquid chromatography with tandem mass spectrometry.

To clarify whether CSE+NRG-EVs contain naringenin, the content of naringenin in CSE+NRG-EVs was determined by HPLC-MS/MS. As shown in **Figure 2**, compared with the naringenin reference standard, the peak height of CSE+NRG-EVs was extremely low, indicating that naringenin was almost absent in CSE+NRG-EVs.

### CSE+NRG-EVs suppressed M1 polarization of RAW264.7 macrophages

**Figure 3:**
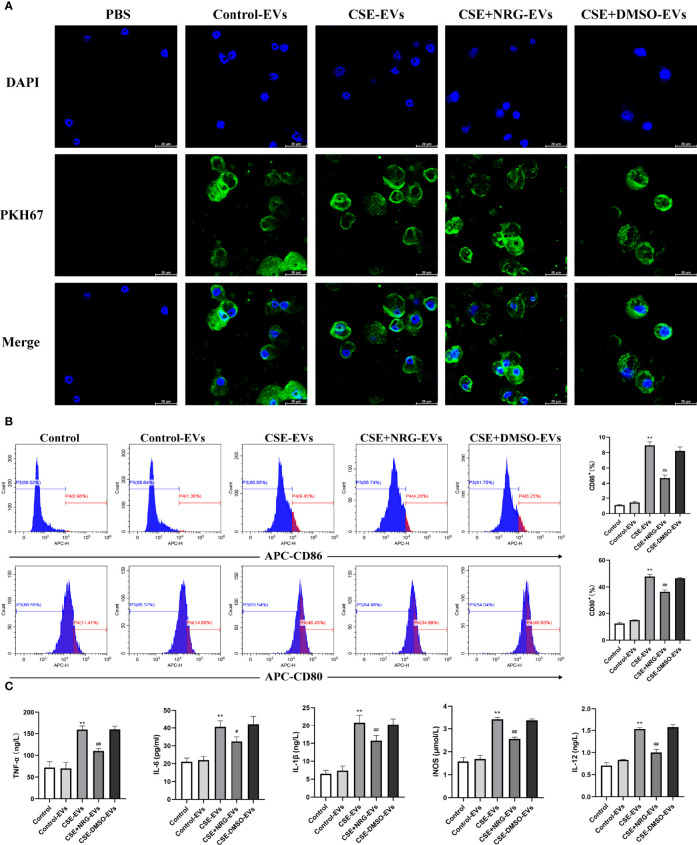
CSE+NRG-EVs inhibited M1 macrophage polarization of RAW264.7 macrophages induced by CSE-EVs. **(A)** Confocal microscopy imaging revealed the uptake of PKH67-labeled EVs by RAW264.7 macrophages. Scale bar = 20 μm. **(B)** Flow cytometry analysis of M1 macrophages (CD86^+^ and CD80^+^) population in RAW264.7 macrophages treated with EVs (n = 3). **(C)** ELISA was performed to evaluate the secretion of M1-associated cytokines including tumor necrosis factor (TNF)-α, interleukin (IL)-6, IL-1β, inducible nitric oxide synthase (iNOS), and IL-12 of RAW264.7 macrophages (n = 3). **p < 0.01 *vs*. Control-EVs group. ^#^ *p* < 0.05, ^##^ *p* < 0.01 *vs*. CSE-EVs group.

RAW264.7 macrophage was considered a typical cell line for studying macrophage function, thus we firstly utilized RAW264.7 macrophages to investigate the role of CSE+NRG-EVs on macrophage polarization. To clarify whether BEAS-2B-derived EVs can be internalized by RAW264.7 macrophages, PKH67-labeled EVs were incubated with RAW264.7 cells for 12 h. Subsequent confocal microscope imaging showed that the green fluorescence dyed EVs were dotted around the blue fluorescence dyed nuclei of macrophages, suggesting that BEAS-2B-derived EVs could be concentrated in the cytoplasm of RAW264.7 macrophages (**Figure 3A**).

Flow cytometry and ELISA were performed to investigate the role of CSE+NRG-EVs on M1 macrophage polarization of RAW264.7 macrophages. In contrast to CSE-EVs, the percentages of M1 phenotype (CD86^+^ and CD80^+^) cells were found to be decreased by the treatment of CSE+NRG-EVs (**Figure 3B**). Furthermore, we observed that the secretion of M1-associated cytokines including TNF-α, IL-6, IL-1β, iNOS, and IL-12 in the CSE+NRG-EVs group was significantly reduced in comparison with that in the CSE-EVs group (**Figure 3C**). These data suggested that CSE+NRG-EVs inhibited M1 polarization of RAW264.7 macrophages induced by CSE-EVs.

### CSE+NRG-EVs suppressed M1 polarization of MH-S macrophages

**Figure 4:**
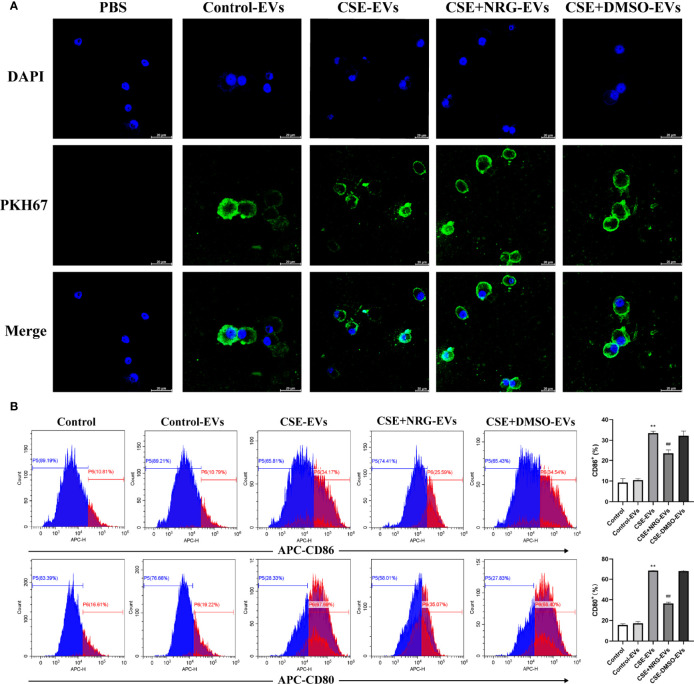
CSE+NRG-EVs suppressed M1 macrophage polarization of MH-S macrophages induced by CSE-EVs. **(A)** Uptake of BEAS-2B-derived EVs by MH-S macrophages imaged by confocal microscopy. Scale bar = 20 μm. **(B)** Representative figures showing the effects of different groups of EVs on M1 polarization. CSE+NRG-EVs significantly decreased the M1 rates of MH-S (n = 3). **p < 0.01 *vs*. Control-EVs group. ^##^ *p* < 0.01 *vs*. CSE-EVs group.

Alveolar macrophages, important immune cells in the lung, are mainly responsible for eliminating invaded pathogens and timely adjusting the immune status to alleviate tissue damage (11). To further investigate the role of CSE+NRG-EVs on M1 macrophage polarization, we analyzed M1-associated markers of MH-S macrophages by flow cytometry. Confocal microscope imaging confirmed that BEAS-2B-derived EVs were taken up by MH-S macrophages (**Figure 4A**). The flow cytometry results showed that CSE+NRG-EVs markedly decreased the ratios of CD86^+^ and CD80^+^ macrophages, indicating that the M1 polarization of MH-S macrophages was inhibited by the treatment of CSE+NRG-EVs (**Figure 4B**). These findings suggested that CSE+NRG-EVs exerted an inhibitory effect on the M1 polarization of MH-S macrophages induced by CSE-EVs.

### CSE+NRG-EVs suppressed M1 polarization of THP-1 macrophages

**Figure 5:**
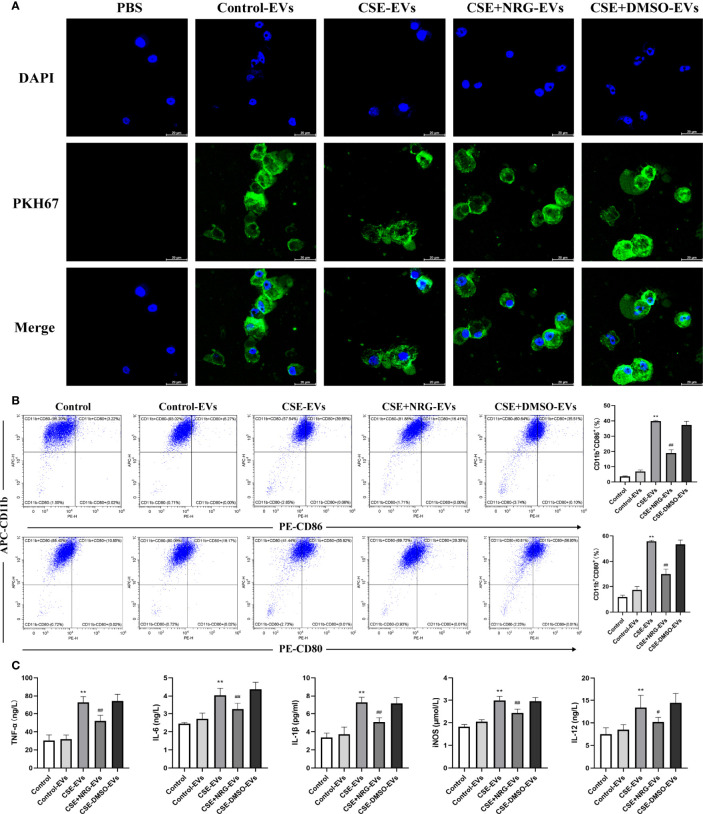
CSE+NRG-EVs inhibited M1 macrophage polarization of THP-1 macrophages induced by CSE-EVs. **(A)** Uptake of BEAS-2B-derived EVs by THP-1 macrophages detected by confocal microscopy. Scale bar = 20 μm. **(B)** Flow cytometry analysis of the percentages of CD86^+^ and CD80^+^ THP-1 macrophages treated with different groups of EVs (n = 3). **(C)** ELISA was performed to measure the secretion levels of TNF-α, IL-6, IL-1β, iNOS, and IL-12 of THP-1 macrophages (n = 6). **p < 0.01 *vs*. Control-EVs group. ^#^ *p* < 0.05, ^##^ *p* < 0.01 *vs*. CSE-EVs group.

Monocytes are a major source of resident macrophages in the lung, especially in the initial phase of inflammation response (12). To clarify if the inhibition of M1 macrophage polarization was also observed in the human macrophage cell line. We incubated the THP-1 macrophages with BEAS-2B-derived EVs and examined the levels of M1-associated macrophage markers. THP-1 monocytes presented as rounded cells and differentiated into spindle-shaped or irregular polygon M0 macrophages by PMA treatment (**Supplementary 2**). Similarly, we observed the internalization of BEAS-2B-derived EVs by THP-1 macrophages utilizing confocal microscopy (**Figure 5A**).

The percentage of polarization-related surface markers of macrophages was detected by flow cytometry (**Figure 5B**). High positivity for CD11b demonstrated that THP-1 monocytes were efficiently differentiated into M0 macrophages in response to PMA. Furthermore, CSE-EVs significantly increased the CD11b^+^CD86^+^ and CD11b^+^CD80^+^ cells percentage of THP-1 macrophages, instead, CSE+NRG-EVs markedly reduced the percentages of CD11b^+^CD86^+^ and CD11b^+^CD80^+^ cells. The concentration of M1-associated cytokines of THP-1 macrophages in the supernatants was measured by ELISA (**Figure 5C**). CSE+NRG-EVs also reversed the secretion of TNF-α, IL-1β, IL-6, iNOS, and IL-12 induced by CSE-EVs in THP-1 macrophages, as the same in RAW264.7 macrophages. Together these results suggested that CSE+NRG-EVs also played an inhibitory role in the M1 polarization of THP-1 macrophages induced by CSE-EVs.

### Naringenin downregulated EVs miR-21-3p which targeted PTEN/AKT pathway thereby suppressing M1 macrophage polarization

**Figure 6:**
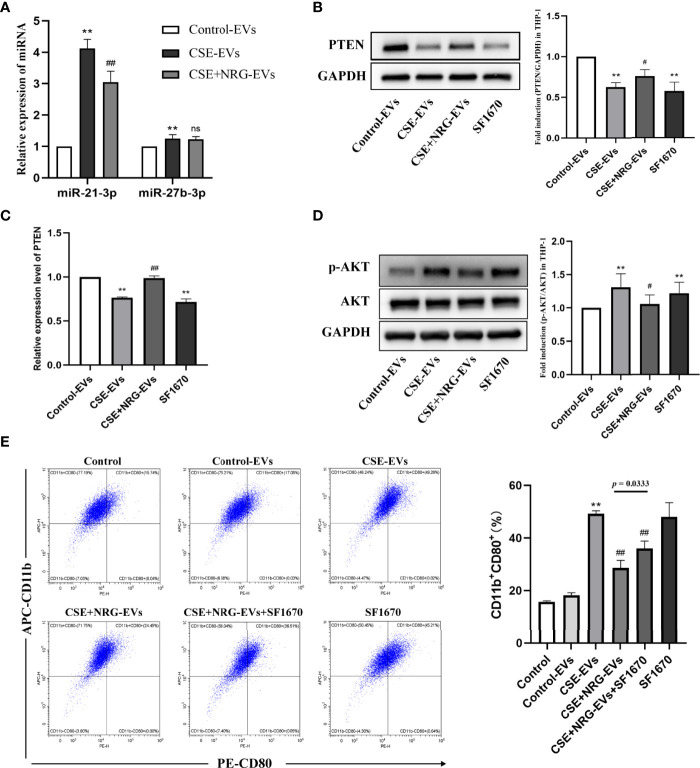
Naringenin downregulated BEAS-2B-derived EVs miR-21-3p which targeted tensin homolog deleted on chromosome ten/protein kinase B (PTEN/AKT) cascade thereby suppressing M1 macrophage polarization. **(A)** Quantitative real-time PCR (qRT-PCR) was performed to detect the expression levels of miR-21-3p and miR-27b-3p in EVs (n = 5). **(B)** Expression of PTEN was quantified using western blot analysis (n = 6). **(C)** The mRNA level of PTEN was determined using qRT-PCR analysis (n = 6). **(D)** Western blotting was utilized for the p-AKT/AKT level (n = 6). **(E)** The CD11b^+^CD80^+^ THP-1 macrophages treated by EVs or SF1670 (PTEN inhibitor) were indicated by flow cytometry (n = 3). **p < 0.01 *vs*. Control-EVs group. ^#^ *p* < 0.05, ^##^ *p* < 0.01 *vs*. CSE-EVs group.

In our previous study, we found that CSE promoted M1 macrophage polarization *via* upregulated EVs miR-21-3p and miR-27b-3p derived from BEAS-2B cells (5). Thus, we speculated that naringenin suppressed M1 macrophage polarization *via* downregulating these miRNAs in this study. QRT-PCR indicated the miR-21-3p level, but not the miR-27b-3p level, in CSE+NRG-EVs was markedly decreased compared with CSE-EVs (**Figure 6A**).

Zhu et al. (13) performed dual-luciferase reporter assays and affirmed that the miR-21-3p targeted the conserved sequence of PTEN 3′-UTR in human cancerous liver cells, which was predicted by TargetScan and PicTar databases. Furthermore, Hu et al. (14) found that transfection of miR-21-3p inhibitors increased the PTEN level in human microvascular endothelial cells. PTEN is a classical suppressor that antagonizes the phosphatidylinositol 3-kinase/protein kinase B (PI3K/AKT) signaling pathway (15). In this study, we examined whether CSE+NRG-EVs targeted the PTEN/AKT pathway of macrophages. Western blotting and qRT-PCR confirmed that the PTEN protein and mRNA levels remarkably decreased in THP-1 macrophages with CSE-EVs treatment, while significantly increasing in the CSE+NRG-EVs group (*vs.* CSE-EVs group) (**Figures 6B, C**). Correspondingly, phosphorylation of AKT/total AKT (p-AKT/AKT) protein level was upregulated by CSE-EVs treatment, while reversed by CSE+NRG-EVs treatment (**Figure 6D**). These findings suggested that naringenin could downregulate BEAS-2B-derived EVs miR-21-3p, thereby increasing the PTEN level and inhibiting the activation of AKT of macrophages.

We then clarified the relation between PTEN and the inhibitory effect of CSE+NRG-EVs on M1 macrophage polarization. Flow cytometry analysis revealed that the ratio of the CD11b^+^CD80^+^ cells in the CSE+NRG-EVs in combination with SF1670 group was significantly higher than in the CSE+NRG-EVs group but lower than in the CSE-EVs group (**Figure 6E**). These results indicated that inhibition of PTEN expression attenuated but did not completely abolish the inhibitory effect of CSE+NRG-EVs on M1 macrophage polarization. Altogether, the outcomes suggested that naringenin could downregulate BEAS-2B-derived EVs miR-21-3p targeting PTEN/AKT cascade in macrophages, which was one of the mechanisms of suppressing M1 macrophage polarization.

### Naringenin downregulating EVs PARP1 thereby suppressing M1 macrophage polarization probably

**Figure 7:**
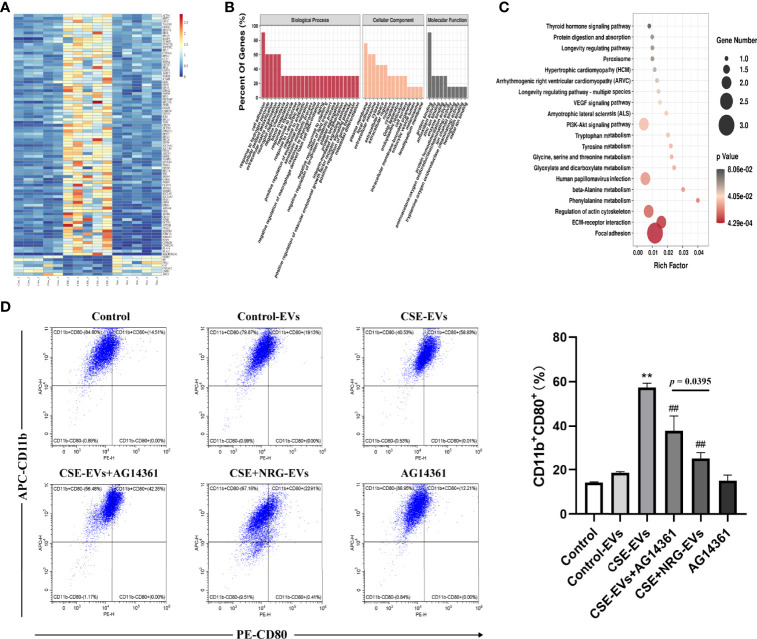
Naringenin decreased BEAS-2B-derived EVs poly ADP-ribose polymerase (PARP)1 expression thereby suppressing M1 macrophage polarization probably. **(A)** The heat map for 91 EVs proteins which were significantly reversely expressed by the treatment of naringenin. Aggregation of the same color blocks indicated proteomics reproducibility (n = 5). **(B)** GO analysis for biological process, cellular component, and molecular function terms were performed on 91 reversed EVs proteins. **(C)** KEGG pathway enrichment analysis for the 91 EVs protein targets. The size of the dot indicates the number of target genes in the pathway, and the color of the dot reflects the different *p*-value. **(D)** Representative flow cytometry data on CD11b^+^CD80^+^ THP-1 macrophages treated by EVs or AG14361 (PARP1 inhibitor) (n = 3). **p < 0.01 *vs*. Control-EVs group. ^##^ *p* < 0.01 *vs*. CSE-EVs group.

Considering that proteins are also essential cargoes in EVs, we performed EVs proteomics to further investigate whether naringenin altered protein profiles of EVs thereby inhibiting M1 macrophage polarization. By nano-LC-MS/MS, 1001 proteins were identified, 208 of which were selected as significantly differential expressions with Fold change > 2 or < 0.5 and *p*-value < 0.05 in CSE-EVs compared to Control-EVs, while 251 differentially expressed proteins were identified in CSE+NRG-EVs compared to CSE-EVs. The heat map illustrated that 91 proteins were significantly reversed by the treatment of naringenin in EVs, including 84 proteins upregulated in CSE-EVs and downregulated in CSE+NRG-EVs, and 7 proteins with the opposite trend (**Figure 7A**). The original proteomic data were shown in **Supplementary 3**.

For the 91 reversed proteins, functional enrichment analysis was performed for the three GO domains “biological process”, “cellular component”, “molecular function” and KEGG signaling pathways. Concerning the biological process, the reversed proteins were involved in the “cell adhesion”, the “extracellular matrix organization”, the “negative regulation of macrophage-derived foam cell differentiation”, and the “positive regulation of gene expression”. Proteins of the cellular component showed association with the “plasma membrane”, the “extracellular exosome”, the “cytoplasm”, the “cell surface”, and the “endocytic vesicle lumen”. Finally, the biological process revealed that the reversed proteins represented the “protein binding”, the “antioxidant activity”, the “zinc ion binding”, the “enzyme binding”, the “protein homodimerization activity” and so on (**Figure 7B**). KEGG pathways were mainly enriched in “the focal adhesion”, the “ECM-receptor interaction”, the “VEGF signaling pathway”, the “PI3K/AKT signaling pathway”, and so on (**Figure 7C**). Enrichment analysis suggested that CSE+NRG-EVs suppressed M1 macrophage polarization might involve these biological processes and signaling pathways.

Existing evidence showed that PARP1 promoted a high glucose-induced phenotype shift in macrophages toward an inflammatory M1 phenotype (16). And in our proteomics, PARP1 was found to be remarkably upregulated in CSE-EVs (*vs*. Control-EVs, *p* = 0.0016) but downregulated in CSE+NRG-EVs (*vs*. CSE-EVs, *p* = 0.0123). Thus, we investigated whether EVs PARP1 was associated with the promotion effects of CSE-EVs on M1 macrophage polarization in the present study. Flow cytometry analysis indicated that the percentage of CD11b^+^CD80^+^ cells in the CSE-EVs in combination with the AG14361 group was significantly lower than in the CSE-EVs group but higher than in the CSE+NRG-EVs group (**Figure 7D**). These outcomes indicated that inhibition of PARP1 expression attenuated but did not eliminate the promoting effect of CSE-EVs on M1 macrophage polarization, suggesting that naringenin could downregulate BEAS-2B-derived EVs PARP1, which was another mechanism of suppressing M1 macrophage polarization probably.

## Discussions

Epitheliums are the main barrier cells against the invasion of pathogens and macrophages are the major immune cells in the lung that determine the resolution of inflammation (17, 18). In particular, macrophage polarization involves and influences the orientation and the magnitude of inflammatory responses (19). A great number of publications have shown that both epitheliums and macrophages play critical roles in cigarette smoke-induced lung diseases such as COPD (20–23). However, the mechanism of EVs-mediated epithelium-macrophage crosstalk has not been elucidated and their roles in cigarette smoke-induced lung diseases are few studied. In our previous study, we found that CSE-treated BEAS-2B-derived EVs promoted M1 macrophage polarization (5).

Inhibition of M1 macrophage polarization seems to be a feasible strategy for the treatment of inflammatory diseases (24). Accessible evidence (7, 25) has proved that naringenin could suppress M1 macrophage polarization to attenuate inflammation, but whether naringenin could alter epithelium-derived extracellular vesicular cargoes thereby regulating macrophage polarization has not been reported. In this study, we firstly found that naringenin and CSE co-treated BEAS-2B-derived EVs significantly inhibited the expression of CD86 and CD80 and the secretion of TNF- α, IL-6, IL-1 β, iNOS, and IL-12 in macrophage. These outcomes indicated that naringenin suppressed M1 macrophage polarization induced by CSE-treated BEAS-2B-derived EVs *via* altering their cargoes.

Accumulating studies showed that PTEN was involved in the process of M1 macrophage polarization. In this study, we found that naringenin downregulated EVs miR-21-3p to activate PTEN in macrophages. And inhibiting PTEN expression attenuated the inhibitory effect of naringenin and CSE co-treated BEAS-2B-derived EVs on M1 macrophage polarization. Furthermore, PTEN could inhibit the AKT activation and the PI3K/AKT pathway possessed a low *p*-value and a high rich factor in the KEGG enrichment. Liu et al. (26) found that mesenchymal stem cell-derived EVs inhibited M1 macrophage polarization by activating the PTEN/AKT pathway, thereby suppressing the inflammatory response in diabetic mice. Kai et al. (27) proved that regulatory T lymphocytes ameliorated intracerebral hemorrhage-induced inflammatory injury by suppressing M1 macrophage polarization through the IL-10/GSK3β/PTEN axis. Together these findings indicated that naringenin downregulated BEAS-2B-derived EVs miR-21-3p which targeted the PTEN/AKT cascade of macrophages and then suppressed M1 macrophage polarization.

EVs can influence the function of recipient cells by delivering information through their contained abundant proteins (28). In the present study, we performed proteomic analysis and found that naringenin decreased EVs PARP1 expression. Further research revealed that inhibition of PARP1 attenuated the promotional effect of CSE-treated BEAS-2B-derived EVs on M1 macrophage polarization. Clinical studies showed that PARP1 activity and mRNA level were significantly higher in COPD patients compared to non-smokers, which were positively correlated with the progression of COPD (29). Choudhuri et al. (30) found that inhibiting PARP1 activity or knocking out PARP1 gene could significantly suppress M1 macrophage polarization. These outcomes suggested that naringenin downregulated BEAS-2B-derived EVs PARP1 and then suppressed M1 macrophage polarization probably.

**Figure 8:**
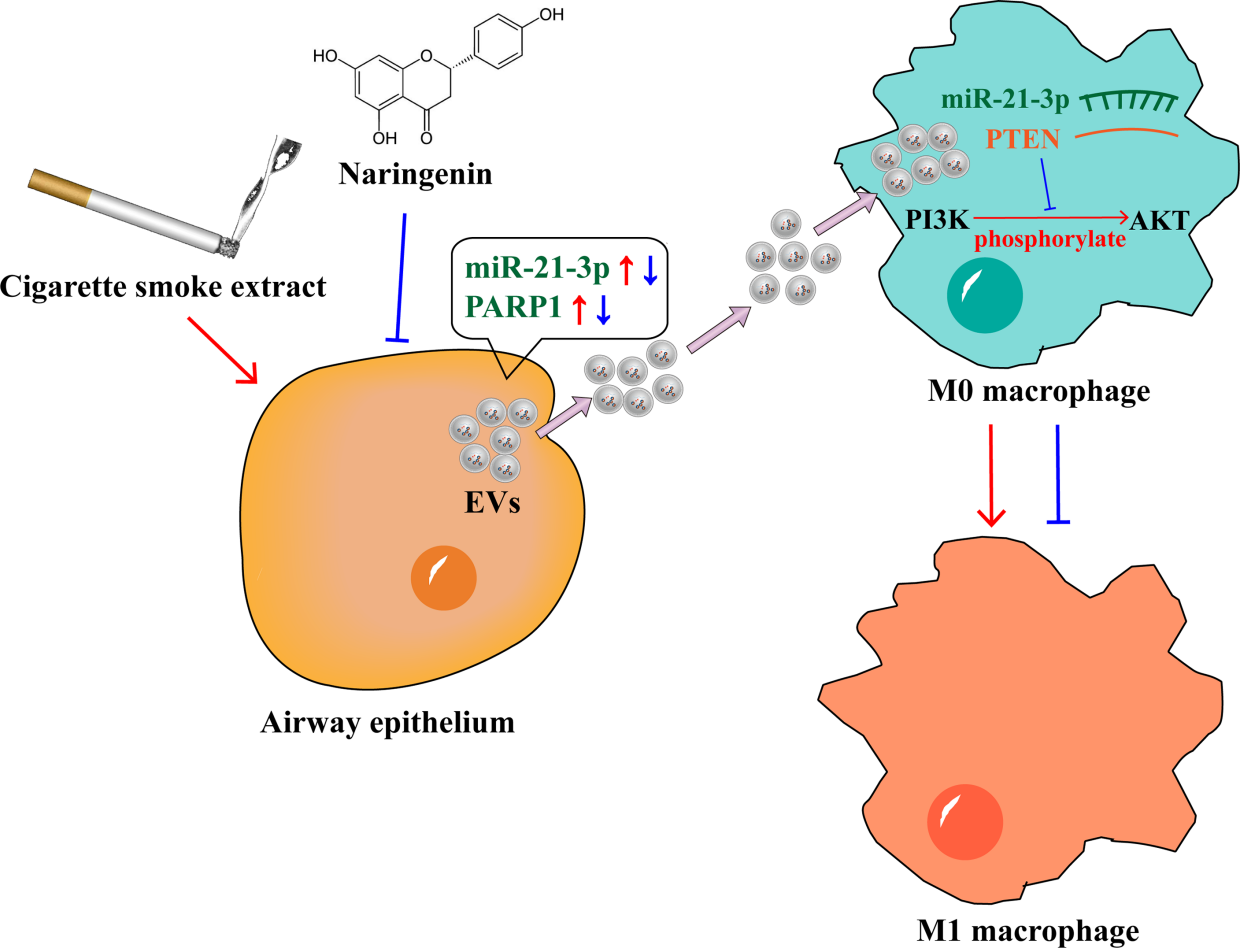
Naringenin suppressed BEAS-2B-derived extracellular vesicular cargoes disorder caused by cigarette smoke extract thereby inhibiting M1 macrophage polarization.

In conclusion, we found that naringenin suppressed BEAS-2B-derived extracellular vesicular cargoes disorder caused by CSE thereby inhibiting M1 macrophage polarization. The underlying mechanisms included that naringenin downregulated BEAS-2B-derived EVs miR-21-3p which targeted PTEN/AKT cascade and naringenin decreased BEAS-2B-derived EVs PARP1 expression, and then suppressed M1 macrophage polarization (**Figure 8**). Indeed, further studies are needed to address several unanswered questions. It is still not clear whether naringenin regulates macrophage polarization to attenuate lung inflammation *in vivo via* altering extracellular vesicular cargoes derived from airway epithelium. Our results also warrant further investigation to discover whether naringenin regulates macrophage polarization *via* altering the other cargoes such as long non-coding RNAs, circular RNAs, and lipids. Furthermore, a limitation of our study is that the macrophage cell lines but not human primary macrophages were used. Therefore, experiments using human primary CD14 positive peripheral blood mononuclear cell-derived macrophages will be worth conducting to demonstrate M1 macrophage polarization in our future investigations. In a word, our study provides novel scientific bases for clarifying the mechanism of naringenin in the treatment of cigarette smoke-induced lung inflammatory diseases.

## Data availability statement

The datasets presented in this study can be found in online repositories. The names of the repository/repositories and accession number(s) can be found below: http://www.proteomexchange.org/, PXD033545.

## Author contributions

PL and WS provided the concept and designed the research. PL and YW supervised the research. ZC, HW, WF, JZ, and YY performed the experiments and collected data. ZC and HW analyzed the data. ZC and PL wrote the manuscript. All authors have read and approved the final manuscript. All authors contributed to the article and approved the submitted version.

## Funding

This work was financially supported by the Applied Science and Technology R&D Special Fund Project of Guangdong Province (No. 2016B020239003) and the Science and Technology Program of Guangdong Province (No. 2019B090905002).

## Conflict of interest

The authors declare that the research was conducted in the absence of any commercial or financial relationships that could be construed as a potential conflict of interest.

## Publisher’s note

All claims expressed in this article are solely those of the authors and do not necessarily represent those of their affiliated organizations, or those of the publisher, the editors and the reviewers. Any product that may be evaluated in this article, or claim that may be made by its manufacturer, is not guaranteed or endorsed by the publisher.

## Supplementary material

Supplementary 1 The list of primer sequences used in this study.

Supplementary 2 Differentiation induction of THP-1 cells. **(A)** Rounded THP-1 monocytes differentiated into **(B)** spindle-shaped or irregular polygon M0 macrophages with the treatment of phorbol ester. Scale bar = 200 μm.

Supplementary 3 The original proteomic data of EVs in this study.

## References

[1] Strzelak A Ratajczak A Adamiec A Feleszko W. Tobacco smoke induces and alters immune responses in the lung triggering inflammation, allergy, asthma and other lung diseases: a mechanistic review. Int J Environ Res Public Health (2018) 15(5):1033. doi: 10.3390/ijerph15051033 PMC5982072 29883409

[2] Mohan A Agarwal S Clauss M Britt NS Dhillon NK. Extracellular vesicles: novel communicators in lung diseases. Respir Res (2020) 21(1):175. doi: 10.1186/s12931-020-01423-y 32641036 PMC7341477

[3] Bissonnette EY Lauzon-Joset JF Debley JS Ziegler SF. Cross-talk between alveolar macrophages and lung epithelial cells is essential to maintain lung homeostasis. Front Immunol (2020) 11:583042. doi: 10.3389/fimmu.2020.583042 33178214 PMC7593577

[4] Shapouri-Moghaddam A Mohammadian S Vazini H Taghadosi M Esmaeili SA Mardani F. Macrophage plasticity, polarization, and function in health and disease. J Cell Physiol (2018) 233(9):6425–40. doi: 10.1002/jcp.26429 PMC13482560 29319160

[5] Chen Z Wu H Shi R Fan W Zhang J Su W. MiRNAomics analysis reveals the promoting effects of cigarette smoke extract-treated beas-2B-derived exosomes on macrophage polarization. Biochem Biophys Res Commun (2021) 572:157–63. doi: 10.1016/j.bbrc.2021.07.093 34365140

[6] Chen Z Chen P Wu H Shi R Su W Wang Y. Evaluation of naringenin as a promising treatment option for COPD based on literature review and network pharmacology. Biomolecules (2020) 10(12):1644. doi: 10.3390/biom10121644 PMC7762561 33302350

[7] Karuppagounder V Arumugam S Thandavarayan RA Sreedhar R Giridharan VV Pitchaimani V. Naringenin ameliorates skin inflammation and accelerates phenotypic reprogramming from M1 to M2 macrophage polarization in atopic dermatitis NC/Nga mouse model. Exp Dermatol (2016) 25(5):404–7. doi: 10.1111/exd.12962 26836240

[8] Xu H Ling M Xue J Dai X Sun Q Chen C. Exosomal microRNA-21 derived from bronchial epithelial cells is involved in aberrant epithelium-fibroblast cross-talk in COPD induced by cigarette smoking. Theranostics (2018) 8(19):5419–33. doi: 10.7150/thno.27876 PMC6276085 30555555

[9] Zhao S Mi Y Guan B Zheng B Wei P Gu Y. Tumor-derived exosomal miR-934 induces macrophage M2 polarization to promote liver metastasis of colorectal cancer. J Hematol Oncol (2020) 13(1):156. doi: 10.1186/s13045-020-00991-2 33213490 PMC7678301

[10] Jia L Zhou X Huang X Xu X Jia Y Wu Y. Maternal and umbilical cord serum-derived exosomes enhance endothelial cell proliferation and migration. FASEB J (2018) 32(8):4534–43. doi: 10.1096/fj.201701337RR 29570394

[11] Joshi N Walter JM Misharin AV. Alveolar macrophages. Cell Immunol (2018) 330:86–90. doi: 10.1016/j.cellimm.2018.01.005 29370889

[12] Hume DA Irvine KM Pridans C. The mononuclear phagocyte system: the relationship between monocytes and macrophages. Trends Immunol (2019) 40(2):98–112. doi: 10.1016/j.it.2018.11.007 30579704

[13] Zhu Y Tang H Zhang L Gong L Wu G Ni J. Suppression of miR-21-3p enhances TRAIL-mediated apoptosis in liver cancer stem cells by suppressing the PI3K/Akt/Bad cascade *via* regulating PTEN. Cancer Manag Res (2019) 11:955–68. doi: 10.2147/CMAR.S183328 PMC6349085 30774424

[14] Hu Y Rao SS Wang ZX Cao J Tan YJ Luo J. Exosomes from human umbilical cord blood accelerate cutaneous wound healing through miR-21-3p-mediated promotion of angiogenesis and fibroblast function. Theranostics (2018) 8(1):169–84. doi: 10.7150/thno.21234 PMC5743467 29290800

[15] Hu M Zhu S Xiong S Xue X Zhou X. MicroRNAs and the PTEN/PI3K/Akt pathway in gastric cancer (Review). Oncol Rep (2019) 41(3):1439–54. doi: 10.3892/or.2019.6962 30628706

[16] Li P Wang Y Liu X Liu B Wang ZY Xie F. Loss of PARP-1 attenuates diabetic arteriosclerotic calcification *via* Stat1/Runx2 axis. Cell Death Dis (2020) 11(1):22. doi: 10.1038/s41419-019-2215-8 31924749 PMC6954221

[17] Hewitt RJ Lloyd CM. Regulation of immune responses by the airway epithelial cell landscape. Nat Rev Immunol (2021) 21(6):347–62. doi: 10.1038/s41577-020-00477-9 PMC7804588 33442032

[18] Shotland AM Fontenot AP McKee AS. Pulmonary macrophage cell death in lung health and disease. Am J Respir Cell Mol Biol (2021) 64(5):547–56. doi: 10.1165/rcmb.2020-0420TR PMC8086041 33332993

[19] Funes SC Rios M Escobar-Vera J Kalergis AM. Implications of macrophage polarization in autoimmunity. Immunology (2018) 154(2):186–95. doi: 10.1111/imm.12910 PMC5980179 29455468

[20] Tatsuta M Kan-O K Ishii Y Yamamoto N Ogawa T Fukuyama S. Effects of cigarette smoke on barrier function and tight junction proteins in the bronchial epithelium: protective role of cathelicidin LL-37. Respir Res (2019) 20(1):251. doi: 10.1186/s12931-019-1226-4 31706310 PMC6842552

[21] Aghapour M Raee P Moghaddam SJ Hiemstra PS Heijink IH. Airway epithelial barrier dysfunction in chronic obstructive pulmonary disease: role of cigarette smoke exposure. Am J Respir Cell Mol Biol (2018) 58(2):157–69. doi: 10.1165/rcmb.2017-0200TR 28933915

[22] Le Y Wang Y Zhou L Xiong J Tian J Yang X. Cigarette smoke-induced HMGB1 translocation and release contribute to migration and NF-κB activation through inducing autophagy in lung macrophages. J Cell Mol Med (2020) 24(2):1319–31. doi: 10.1111/jcmm.14789 PMC6991703 31769590

[23] Lugg ST Scott A Parekh D Naidu B Thickett DR. Cigarette smoke exposure and alveolar macrophages: Mechanisms for lung disease. Thorax (2022) 77(1):94–101. doi: 10.1136/thoraxjnl-2020-216296 33986144 PMC8685655

[24] Wang Y Smith W Hao D He B Kong L. M1 and M2 macrophage polarization and potentially therapeutic naturally occurring compounds. Int Immunopharmacol (2019) 70:459–66. doi: 10.1016/j.intimp.2019.02.050 30861466

[25] Mendes LF Gaspar VM Conde TA Mano JF Duarte IF. Flavonoid-mediated immunomodulation of human macrophages involves key metabolites and metabolic pathways. Sci Rep (2019) 9(1):14906. doi: 10.1038/s41598-019-51113-z 31624286 PMC6797761

[26] Liu W Yu M Xie D Wang L Ye C Zhu Q. Melatonin-stimulated MSC-derived exosomes improve diabetic wound healing through regulating macrophage M1 and M2 polarization by targeting the PTEN/AKT pathway. Stem Cell Res Ther (2020) 11(1):259. doi: 10.1186/s13287-020-01756-x 32600435 PMC7322868

[27] Zhou K Zhong Q Wang YC Xiong XY Meng ZY Zhao T. Regulatory T cells ameliorate intracerebral hemorrhage-induced inflammatory injury by modulating microglia/macrophage polarization through the IL-10/GSK3β/PTEN axis. J Cereb Blood Flow Metab (2017) 37(3):967–79. doi: 10.1177/0271678X16648712 PMC5363473 27174997

[28] Zhang Y Bi J Huang J Tang Y Du S Li P. Exosome: a review of its classification, isolation techniques, storage, diagnostic and targeted therapy applications. Int J Nanomedicine (2020) 15:6917–34. doi: 10.2147/IJN.S264498 PMC7519827 33061359

[29] Hageman GJ Larik I Pennings HJ Haenen GR Wouters EF Bast A. Systemic poly(ADP-ribose) polymerase-1 activation, chronic inflammation, and oxidative stress in COPD patients. Free Radic Biol Med (2003) 35(2):140–8. doi: 10.1016/s0891-5849(03)00237-5 12853070

[30] Choudhuri S Garg NJ. PARP1-cGAS-NF-κB pathway of proinflammatory macrophage activation by extracellular vesicles released during trypanosoma cruzi infection and chagas disease. PloS Pathog (2020) 16(4):e1008474. doi: 10.1371/journal.ppat.1008474 32315358 PMC7173744

