# Supplementary material for: Naringenin suppresses BEAS-2B-derived extracellular vesicular cargoes disorder caused by cigarette smoke extract thereby inhibiting M1 macrophage polarization
Source: Front Immunol. 2022 Jul 18;13:930476. doi: 10.3389/fimmu.2022.930476 (PMC9342665; doi:10.3389/fimmu.2022.930476)
Supplement: Supplementary 1 — The list of primer sequences used in this study. [file Table_2.docx]

| Name | Forward primer（5' - 3'） | Reverse primer（5' - 3'） |
| --- | --- | --- |
| miR-21-3p | GCTTATCAGACTGATGTTG | \ |
| miR-27b-3p | GAGCTTAGCTGATTGGTG | \ |
| U6 | GCTTCGGCAGCACATATACTAAAAT | \ |
| PTEN | TTTGATGCTGCCGGTAAACTCCACT | CTTACAGTTGGGCCCTGTACCATCC |
| GADPH | GTCTCCTCTGACTTCAACAGCG | ACCACCCTGTTGCTGTAGCCAA |

**Sup.2** The list of primer sequences used in this study
