# Supplementary figures and images for: Naringenin suppresses BEAS-2B-derived extracellular vesicular cargoes disorder caused by cigarette smoke extract thereby inhibiting M1 macrophage polarization
Source: Front Immunol. 2022 Jul 18;13:930476. doi: 10.3389/fimmu.2022.930476 (PMC9342665; doi:10.3389/fimmu.2022.930476)

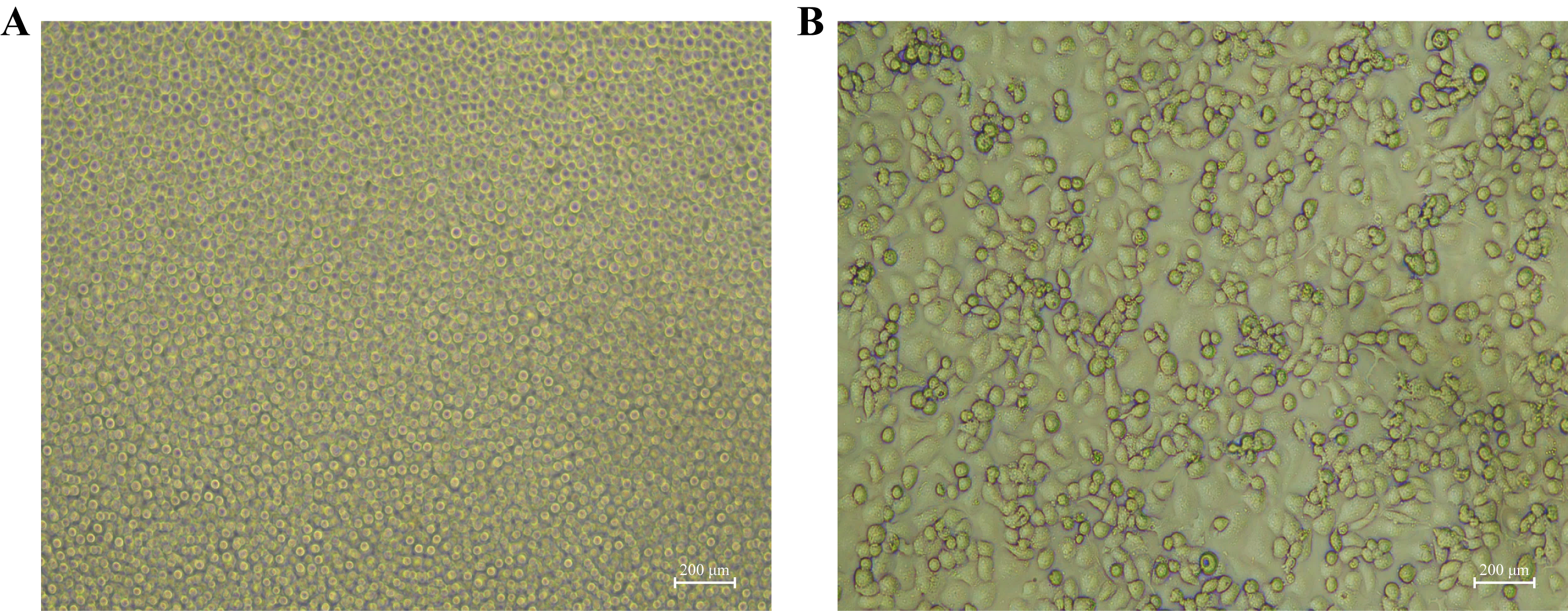

Supplement: Supplementary 2 — Differentiation induction of THP-1 cells. (A) Rounded THP-1 monocytes differentiated into (B) spindle-shaped or irregular polygon M0 macrophages with the treatment of phorbol ester. Scale bar = 200 μm. [file Image_1.tif]
